# Twist-to-bend ratio: an important selective factor for many rod-shaped biological structures

**DOI:** 10.1038/s41598-019-52878-z

**Published:** 2019-11-20

**Authors:** Steve Wolff-Vorbeck, Max Langer, Olga Speck, Thomas Speck, Patrick Dondl

**Affiliations:** 1 Department of Applied Mathematics, University of Freiburg, Hermann-Herder-Str. 10, D-79104 Freiburg, Germany; 2 Plant Biomechanics Group, Botanic Garden, Faculty of Biology, University of Freiburg, Schänzlestraße 1, D-79104 Freiburg, Germany; 3 Cluster of Excellence livMatS @ FIT – Freiburg Center for Interactive Materials and Bioinspired Technologies, University of Freiburg, Georges-Köhler-Allee 105, D-79110 Freiburg, Germany; 4 Freiburg Materials Research Center (FMF), University of Freiburg, Stefan-Meier-Str. 21, D-79104 Freiburg, Germany

**Keywords:** Biomaterials, Plant sciences, Plant evolution, Computational methods, Applied mathematics, Computational science

## Abstract

Mechanical optimisation plays a key role in living beings either as an immediate response of individuals or as an evolutionary adaptation of populations to changing environmental conditions. Since biological structures are the result of multifunctional evolutionary constraints, the dimensionless twist-to-bend ratio is particularly meaningful because it provides information about the ratio of flexural rigidity to torsional rigidity determined by both material properties (bending and shear modulus) and morphometric parameters (axial and polar second moment of area). The determination of the mutual contributions of material properties and structural arrangements (geometry) or their ontogenetic alteration to the overall mechanical functionality of biological structures is difficult. Numerical methods in the form of gradient flows of phase field functionals offer a means of addressing this question and of analysing the influence of the cross-sectional shape of the main load-bearing structures on the mechanical functionality. Three phase field simulations were carried out showing good agreement with the cross-sections found in selected plants: (i) U-shaped cross-sections comparable with those of *Musa* sp. petioles, (ii) star-shaped cross-sections with deep grooves as can be found in the lianoid wood of *Condylocarpon guianense* stems, and (iii) flat elliptic cross-sections with one deep groove comparable with the cross-sections of the climbing ribbon-shaped stems of *Bauhinia guianensis*.

## Introduction

## Biological Materials Systems

During ontogeny and phylogeny, living organisms are confronted with the challenge of immediate individual response and evolutionary adaptation of populations in order to exist within changing environmental conditions^1^ and, simultaneously, have to ensure the survival of the species through reproduction. The German zoologist Günther Osche addressed this dilemma and pointed out that living beings cannot post a sign with the message “closed for reconstruction”^2^. On the contrary, regardless of the respective time scale, responses and adaptations in living organisms must take place during “ongoing operation”, because the fulfilment of life-ensuring functions must be maintained permanently over the entire period. These responses and adaptations can include local or general changes in metabolism and changes in morphological-anatomical characteristics and mechanical properties realised at various hierarchical levels^3^. Because of their hierarchical structure from the molecular to the macroscopic level, a clear differentiation between “material” and “structure” is not possible in biology^4^. On the basis of these smooth transitions Wegst *et al*.^5^ coined the term “structural materials” to describe the complex materials systems of living nature. In other words, plants and animals are materials systems that emerge characteristics far beyond those of their individual components^6^.

From a mechanical point of view, biological materials systems are characterised, on the one hand, by anatomical heterogeneity through a specific three-dimensional arrangement of various tissues and, on the other hand, by mechanical anisotropy through various mechanical properties of their individual tissues. With regard to the topic of this article, response and adaptation can therefore be considered as a consequence of successive or simultaneous changes in one or both of these aspects, which might occur during both ontogeny and phylogeny.

## The Twist-to-Bend Ratio

In the context of response and adaptation to existing or changing environmental mechanical conditions, the dimensionless twist-to-bend ratio is particularly useful as it provides information about the ratio of flexural rigidity to torsional rigidity of materials systems determined by both material properties (bending modulus *E* and shear modulus *G*) and morphometric parameters (axial second moment of area *I* and polar second moment of area *J*). Additionally, it allows a comparison of bodies of different sizes because of its dimensionlessness^7–10^. Flexural rigidity (=bending stiffness = *EI*) and torsional rigidity (=torsional stiffness = *GJ*) describe the resistance of a body to deformation caused by bending or torsion loading in the linear-elastic range. Since both are composite variables that combine material properties and morphometric parameters, they are well suited for quantifying the mechanical functionality of biological and technical structures^7^. On the one hand, sufficient flexural rigidity is relevant to counteract gravity. In plants, this prevents, for example, the sagging of the leaf blades or ensures an upright growth of the stems and thus an advantageous positioning of leaves, flowers and fruits. On the other hand, a low torsional rigidity may help for planar plant organs to streamline themselves under wind loads, e.g. by turning (large) leaves into the wind or by clustering compound leave blades and thus reducing their cross-sectional area and thus ultimately the drag force^7,11,12^.

In order to identify common patterns in the relationship between flexural rigidity and torsional rigidity, Etnier^10^ created a so-called stiffness mechanospace. By mapping the theoretical expectations of ideal beams based on a cross-sectional shape (elliptic, circular) and various Poisson’s ratios varying from 0 to 0.5, biological beams are generally limited to particular regions of the mechanospace. Vogel^7^ reported that elongated biological structures can achieve higher values for *EI*/*GJ* than ideal isotropic and isovolumetric circular solid cylinders with a value of 1.5 (if *E*/*G* is set to 3.0), as natural structures are anatomically inhomogeneous and mechanically anisotropic. In addition to circular and elliptical cross-sectional shapes, square-shaped, triangular and even U-shaped cross-sections exist in biology. For instance, an average *EI*/*GJ* value of 13.3 ± 1.0 has been reported for the hollow and lenticular flower stalks of daffodils (*Narcissus pseudonarcissus*)^13^. Furthermore, the values of the twist-to-bend ratio of the square-shaped stems of *Leonurus cardiaca* range on average between 15 and 19^14^ and the triangular flower stalks of the sedge *Carex acutiformis* lie in the range of 22 and 51^15^. The U-shaped cross-sections of banana petioles (*Musa textilis*) with *EI*/*GJ* values ranging from 40 to 100 show the highest values of any natural structures tested to date^9,11^.

## Aim of the Study

In principle, the dimensionless twist-to-bend ratio is a highly suitable parameter for the analysis and comparison of rod-shaped biological and technical materials systems among and with each other. The aim of this study has been to investigate the development and interrelationship of flexural rigidity and torsional rigidity in relation to cross-sectional shapes of the main load-bearing structural elements by using a mathematical model and suitable simulations. The results of these simulations have then been compared with the load-bearing structural elements (e.g., lignified strengthening tissues such as xylem, vascular bundles or sclerenchyma and collenchyma fibres) in cross-sections of selected biological plant models. These biological models, which have previously been described in the literature, include the leaf stalks of banana plants (*Musa* sp.) with the highest twist-to-bend ratio known to date^9,11^ and the stems of two different lianas (*Condylocarpon guianense*, *Bauhinia guianensis*) with twist-to-bend ratios markedly changing during ontogeny^16^.

The study is divided into three parts: (i) the mathematical model, which is based on a phase field description of the plant stem cross-section in the design space given by a unit square; (ii) the optimisation of the twist-to-bend ratio of the phase field with respect to its geometry by using a gradient flow method and by weighting flexural rigidity (maximal or minimal), torsional rigidity or both factors as objectives for maximisation and minimisation; (iii) a comparison of the phase field shapes and their mechanical properties acquired during the optimisation process with the selected biological plant models and an interpretation of the insights gained.

The three above-mentioned weighting factors (maximal flexural rigidity, minimal flexural rigidity and torsional rigidity) theoretically allow for a large number of diverse simulations. In the context of this study, the authors have selected three exemplary simulations: (i) minimisation of the torsional rigidity and maximisation of the minimal flexural rigidity, (ii) only minimisation of the torsional rigidity and comparison with the case, whereby the maximal flexural rigidity is also minimised, (iii) minimisation of the torsional rigidity and minimisation of the minimal flexural rigidity and comparison with the case, whereby the maximal flexural rigidity is also maximised.

## Mathematical Model

### Plant stems as slender elastic rods

We describe a plant stem as a long thin elastic rod with domain $B=A\times (0,L)$ of length *L* and constant cross-section *A* for an open bounded sufficiently regular domain $A\subset {{\mathbb{R}}}^{2}$. We assume $L\gg {\rm{diam}}A$ as well as material isotropy. It is, of course, possible to take into account heterogeneity and anisotropy of the material when optimising the rigidity properties of cross-sectional shapes, see, e.g.^17–20^. Here, however, we neglect such heterogeneity and anisotropic effects as well as viscosity and other time-dependent processes, since we are only interested in the influence of the cross-sectional shape on the mechanical properties of the stem.

**Figure 1:**
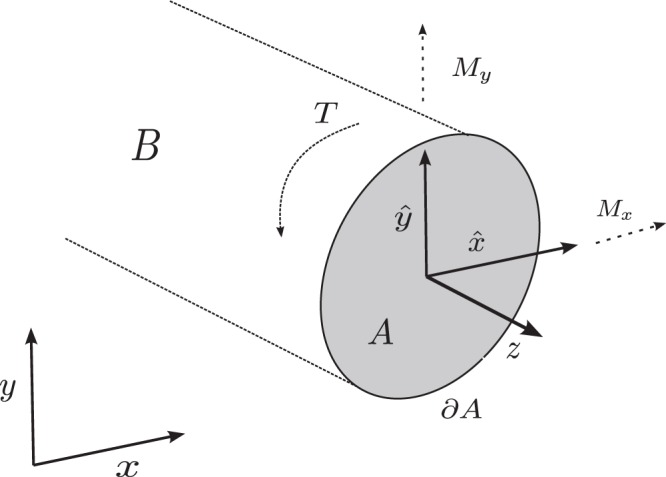
Slender elastic beam *B* subject to bending and torsional moments.

Consider *B* fixed at $z=0$ and bending of *B* to be due to an outer normal force on *A* at $z=L$. Starting from 3D elasticity, in the limit of a slender rod, following Mora & Müller^21^, the flexural (or bending) rigidity is given by the moment curvature relation

$$(\begin{array}{l}{M}_{y}\\ {M}_{x}\end{array})=E(\begin{array}{ll}{D}_{x} & {D}_{xy}\\ {D}_{xy} & {D}_{y}\end{array})\cdot (\begin{array}{l}{\kappa }_{x}\\ {\kappa }_{y}\end{array}),$$

where *M*_*y*_, *M*_*x*_ denote the bending moments on the end of the beam and ${\kappa }_{x}$, ${\kappa }_{y}$ denote the curvature in the direction of *x* and *y*, respectively, see Fig. 1. In our idealised case the bending modulus of elasticity is equivalent to the tensile modulus (Young’s modulus) or compressive modulus of elasticity. Thus the parameter *E* is just the Young’s modulus of the linearisation of the elastic energy and the moments of inertia *D*_*x*_, *D*_*y*_ as well as the product of inertia *D*_*xy*_ are given by

$${D}_{y}=\mathop{\int }\limits_{A}\,{\hat{y}}^{2}\,{\rm{d}}x{\rm{d}}y,\,{D}_{x}=\mathop{\int }\limits_{A}\,{\hat{x}}^{2}\,{\rm{d}}x{\rm{d}}y,\,{D}_{xy}=\mathop{\int }\limits_{A}\,\hat{x}\hat{y}\,{\rm{d}}x{\rm{d}}y,$$

where we have

$$\hat{y}=y-\frac{1}{|A|}\,\mathop{\int }\limits_{A}\,y\,{\rm{d}}x{\rm{d}}y,\,\hat{x}=x-\frac{1}{|A|}\,\mathop{\int }\limits_{A}\,x\,{\rm{d}}x{\rm{d}}y.$$

The maximal and minimal flexural rigidities *D*_max_ and *D*_min_ along the principal axes are then given by the maximal and minimal eigenvalue of the matrix

$$D=(\begin{array}{ll}{D}_{x} & {D}_{xy}\\ {D}_{xy} & {D}_{y}\end{array}),$$

after multiplying with the material Young’s modulus *E*, which leads to

$${D}_{{\rm{\max }}/{\rm{\min }}}=E(\frac{{D}_{x}+{D}_{y}}{2}\pm \sqrt{\frac{{({D}_{x}-{D}_{y})}^{2}}{4}+{D}_{xy}^{2}}).$$

For simplicity we write ${D}_{{\rm{\max }}/{\rm{\min }}}={D}_{{\rm{mean}}}\pm RM$.

*Remark*. Note that Mora & Müller^21^ adapt their coordinate axes to the domain such that $x=\hat{x}$, $y=\hat{y}$, and ${D}_{xy}=0$. Since we will shortly move to a phase field description of the stem cross-section, we will work with arbitrary coordinate axis and origin and thus carry those additional terms.

As for the flexural rigidity, the torsional rigidity for an elastic slender rod with domain *B* was rigorously derived by Mora & Müller^21^. This rigorous derivation considers the limit of a very slender and long rod, for which it is shown that the requirements of St. Venant’s torsion theory^22^ are satisfied. We assume that torsion is due to a moment *T* at the top of *B*, see Fig. 1. Note that for very thin-walled structures (once the thinness of the walls becomes comparable to the length-to-cross-section ratio), the assumptions of St. Venant’s theory of torsion are not applicable and in this case Vlasov’s theory of torsion should be applied. We argue, however, that the structures used in the comparison to plant morphology as displayed in section “Numerical results and comparison to plant morphology” are still within the realm where St. Venant’s theory can be justified.

In this framework the torsional rigidity may be expressed by Prandtl’s stress function. In Prandtl’s stress formulation the shear stress components are described by the derivatives of the stress function $\varphi (x,y)$

$${\sigma }_{zx}=\frac{\partial \varphi (x,y)}{\partial x}\,{\rm{and}}\,{\sigma }_{zy}=-\,\frac{\partial \varphi (x,y)}{\partial y}.$$

Assuming without loss of generality a constant unit twist rate, the stress function $\varphi$ must then satisfy Poisson’s equation

1 $$-\Delta \varphi =2G\,{\rm{on}}\,A,$$

with shear modulus *G*. Using that moments are only appearing on the top of *B*, the traction-free beam wall condition leads to the boundary condition

$$\frac{d\varphi }{ds}=0\,{\rm{on}}\,\partial A\Rightarrow \varphi =const.\,{\rm{on}}\,\partial A,$$

where the boundary $\partial A$ is given by a curve parameterised by *s*.

We restrict ourselves to simply connected plant stem cross-sections and thus may assume, without loss of generality, that

$$\varphi =0\,{\rm{on}}\,\partial A.$$

The torsional rigidity *D*_*z*_ is then given by

$${D}_{z}=2\,\mathop{\int }\limits_{A}\,\varphi \,{\rm{d}}x{\rm{d}}y.$$

### A priori bounds on the twist-to-bend ratio

In a shape optimisation problem regarding the twist-to-bend ratio of a plant stem, we are considering the minimisation problem

2 $$\mathop{{\rm{\inf }}}\limits_{A\subset {{\mathbb{R}}}^{2}}\,{\sigma }_{1}{D}_{z}(A)+{\sigma }_{2}{D}_{{\rm{\min }}}(A)+{\sigma }_{3}{D}_{{\rm{\max }}}(A)$$

with weighting factors ${\sigma }_{1},{\sigma }_{2},{\sigma }_{3}\in {\mathbb{R}}$. For example, if ${\sigma }_{1} > 0$, ${\sigma }_{2} < 0$, and ${\sigma }_{3}=0$, then solutions of Eq. (2) tend to minimise torsional rigidity and maximise minimal flexural rigidity.

Following Kim & Kim^23^ we deduce that *D*_*z*_ has the representation

3 $$\begin{array}{rcl}{D}_{z} & = & \mathop{\int }\limits_{A}\,G({x}^{2}+{y}^{2})\,{\rm{d}}x{\rm{d}}y-\mathop{\int }\limits_{A}\,G[{(\frac{\partial \omega }{\partial x})}^{2}+{(\frac{\partial \omega }{\partial y})}^{2}]\,{\rm{d}}x{\rm{d}}y\\  & = & {(1+\nu )}^{-1}\,{D}_{{\rm{mean}}}-\mathop{\int }\limits_{A}\,G[{(\frac{\partial \omega }{\partial x})}^{2}+{(\frac{\partial \omega }{\partial y})}^{2}]\,{\rm{d}}x{\rm{d}}y,\end{array}$$

where the so-called “warping” function $\omega$ is given by the solution of Laplace’s equation with Neumann boundary condition $\frac{\partial \omega }{\partial \eta }=\frac{1}{2}\frac{d}{ds}|(x(s),y(s)){|}^{2}$, where $(x(s),y(s))$ is an arc-length parametrisation of the boundary curve of the cross-section and $\eta$ is its outer unit normal. This leads to the observation that for a circular domain *A* torsional rigidity *D*_*z*_ is determined by *D*_mean_.

We thus deduce, that, if for example the cross-section *A* is constrained to be a material distribution in a rotationally symmetric reference domain, merely maximising the flexural rigidity leads to non-simply connected domains, such as symmetric hollow circular tubes (see *Condylocarpon guianense*). Therefore, as we are interested in simply connected cross-sections having a high twist-to-bend ratio, a sole maximisation of the flexural rigidity is not useful for our purposes.

For domains with circular boundary curve (simply connected or not) and isotropic material as well as Poisson’s ratio $\nu \in (0,0.5)$ we deduce from Eq. (3) the estimate

$$\frac{{D}_{{\rm{\max }}}}{{D}_{z}}=\frac{{D}_{{\rm{\min }}}}{{D}_{z}}=\frac{{D}_{{\rm{mean}}}}{{D}_{z}}=(1+\nu ) < \frac{3}{2}.$$

The theorem of St. Venant, see, e.g., Pólya^24^, states, that circular domains lead to maximal torsional rigidities among simply connected domains. As a bound for *D*_*z*_(*A*) we can furthermore use the radius ${\rho }_{A}$ of the largest inscribed circle in a domain *A*. This is due to a theorem of Makai^25^, proving the inequality

$${D}_{z}(A)\le 4{\rho }_{A}^{2}|A|$$

for every simply connected domain $A\subset {{\mathbb{R}}}^{2}$.

While it is instructive to consider such bounds on the functional above, the problem in Eq. (2) is ill posed even among simply connected domains, in the sense that no minimum exists. This can easily be seen due to the fact that thin fingers cause high flexural rigidity but no torsional rigidity. Thus, a sequence of thinner, but increasingly wide I-beams will lead to larger and larger negative values of the functional in Eq. (2) for ${\sigma }_{1} > 0$, ${\sigma }_{2} < 0$, ${\sigma }_{3}=0$. We therefore restrict ourselves to a bounded domain for our designs and add a perimeter penalty. Such a perimeter penalisation is indeed also sensible in our application, as plants should not have arbitrary large surfaces of exposure. Furthermore, we impose a fixed cross-sectional area (or, equivalently, mass).

More importantly, however, we are not really interested in the minimisers of the functional themselves. Instead, we consider a gradient flow dynamics of our functional using an artificial time variable, hereafter called pseudo-time. Solutions of this gradient flow are driven towards the direction of maximal decline, i.e., the direction of the biggest change in rigidities for small changes in shape. We propose that shape change in such a direction can be observed in plant stem geometries.

### Phase field approximation

In order to treat our shape optimisation problem numerically, we describe the material distribution in a given domain $\Omega$ by a phase field variable *u*. The phase field *u* shall take values close to 0 in the void and values close to 1 in the areas where material is present. In a phase field approach the interface between material and void is given by a diffuse interface layer, whose thickness is proportional to a small length scale parameter $\varepsilon$. At this interface the phase field smoothly but rapidly changes its value between 0 and 1. The aforementioned mass constraint now simply reads

$$\frac{1}{|\Omega |}\,\mathop{\int }\limits_{\Omega }\,u=m\in (0,1).$$

Further, we assume that $u=0$ on the boundary $\partial \Omega$ of $\Omega$. We use the common approach of Blank *et al*.^26^ to describe the phase transition from material to void in the Young’s modulus *E*, obtaining a Young’s modulus $E(u)$. In our case we simply take

$$E(u)=Eu$$

with material constant *E*. This way we obtain *u*-dependent flexural rigidities ${D}_{{\rm{\max }}/{\rm{\min }}}(u)$ and torsional rigidity ${D}_{z}(u)$. For simplicity, we do not explicitly denote the dependence of these quantities on the length scale $\varepsilon$. Using the model from section “Plant stems as slender elastic rods” the moments of inertia and the product of inertia can now be expressed in terms of

$${D}_{x}(u)=E\,\mathop{\int }\limits_{\Omega }\,{\hat{x}}^{2}u\,{\rm{d}}x{\rm{d}}y,\,{D}_{y}(u)=E\,\mathop{\int }\limits_{\Omega }\,{\hat{y}}^{2}u\,{\rm{d}}x{\rm{d}}y,\,{D}_{xy}(u)=E\,\mathop{\int }\limits_{\Omega }\,\hat{x}\hat{y}u\,{\rm{d}}x{\rm{d}}y,$$

with

$$\hat{x}=x-\frac{1}{m}\,\mathop{\int }\limits_{\Omega }\,xu\,{\rm{d}}x{\rm{d}}y,$$

and

$$\hat{y}=y-\frac{1}{m}\,\mathop{\int }\limits_{\Omega }\,yu\,{\rm{d}}x{\rm{d}}y.$$

In an analogous way we obtain the torsional rigidity ${D}_{z}(u)$ by

$${D}_{z}(u)=2\,\mathop{\int }\limits_{\Omega }\,\varphi (u)\,{\rm{d}}x{\rm{d}}y.$$

If *u* were simply the characteristic function ${\chi }_{A}$ of our plant stem cross-section *A*, then $\varphi (u)$ is given as the solution of Poisson’s problem

4 $$\begin{array}{ll}-\Delta \varphi =2G & {\rm{in}}\,\{u=1\},\\ \varphi =0 & {\rm{on}}\,\partial \{u=1\}.\end{array}$$

In a phase field approach we instead introduce a penalty such that the function $\varphi$ is required to be constant where *u* is close to zero. By choosing zero boundary conditions on $\partial \Omega$, these then get propagated such that $u=0$ on $\Omega \backslash \{u\approx 1\}$. We thus solve

5 $$\begin{array}{rcl}\nabla \cdot (\frac{1}{{(u+{\theta }_{0})}^{2}}\nabla \varphi ) & = & 2G\,{\rm{on}}\,\Omega \\ \varphi  & = & 0\,{\rm{on}}\,\partial \Omega ,\end{array}$$

where $0 < {\theta }_{0}\ll 1$ is a small parameter. As long as the set where $u\approx 1$ is simply connected, we obtain $\varphi$ as an approximation of Prandtl’s stress function in (1). We note that a similar approach, outside of the phase field context, was used by Kim & Kim^23^.

As described before, shape optimisation problems of this kind are in general ill posed and it is necessary to add a perimeter penalisation for regularisation. In phase field approaches such a perimeter penalisation is modelled by the help of the Ginzburg-Landau (or Modica-Mortola) energy^27^

$${{\rm{Per}}}_{\varepsilon }(u)=\frac{1}{{c}_{0}}\,\mathop{\int }\limits_{\Omega }\,\frac{\varepsilon }{2}|\nabla u{|}^{2}+\frac{1}{\varepsilon }F(u)\,{\rm{d}}x{\rm{d}}y,$$

where the function *F* is given by

$$F(u)=\frac{1}{4}{u}^{2}{(u-1)}^{2},$$

so that *F* has exactly two global minima in 0 and 1. The factor $\frac{1}{{c}_{0}}$ is a normalising constant.

We note that with $\varepsilon$ tending to 0 minimisers of ${{\rm{Per}}}_{\varepsilon }(u)$ develop interfaces separating regions in which *u* is nearly constant with values close to the minima of *F*. This is due to an argument by Modica, Theorem I in Modica^28^, which also proves the $\Gamma$-convergence of ${{\rm{Per}}}_{\varepsilon }(u)$ to the perimeter functional ${\rm{Per}}(\{u=1\})$. Thus, adding ${{\rm{Per}}}_{\varepsilon }(u)$ to our problem penalises the perimeter of the set $\{u=1\}$ and hence the perimeter of our cross-section.

The shape optimisation problem is then to find a solution

$$u\in {\mathscr{A}}\{q\in {H}_{0}^{1}(\Omega ):0\le q\le 1\,{\rm{in}}\,\Omega ,\,{\int }^{}\,q=m\}$$

of the following minimisation problem

6 $$\mathop{{\rm{\inf }}}\limits_{u\in {\mathscr{A}}}\,{I}_{\varepsilon }(u)$$

with

$${I}_{\varepsilon }(u)={\sigma }_{1}{D}_{z}(u)+{\sigma }_{2}{D}_{{\rm{\min }}}(u)+{\sigma }_{3}{D}_{{\rm{\max }}}(u)+\gamma {{\rm{Per}}}_{\varepsilon }(u).$$

The function space ${H}_{0}^{1}$ denotes all functions with square integrable derivatives and zero boundary conditions on the boundary of $\Omega$. We note that Eq. (6) does indeed admit minimisers as long as $\gamma  > 0$ and $\Omega$ is bounded.

### *L*^2^-Gradient flow and numerical implementation

To compute solutions of Eq. (6) numerically we use a steepest descent approach, i.e., we make small steps in *u* towards the direction of maximal negative change of ${I}_{\varepsilon }$. In other words, we compute a time-discrete *L*^2^-gradient flow of ${I}_{\varepsilon }$ until a stationary state has been reached using a discretisation of our reference domain by P1 triangular finite elements. Time discretisation uses a time step variable $\tau$ and along with integer iteration steps $n\ge 1$ this leads to an artificial time variable $t=\tau \cdot n$, also called pseudo-time. A thus computed stationary state of the gradient is usually a local solution of our minimisation problem. Furthermore, we can decouple the solution of Poisson’s problem in Eq. (5) from the gradient flow and calculate it separately using a P1 finite element approach. The mass constraint is imposed using a Lagrange multiplier. For an initial configuration *u*^0^ of the phase field variable we use a semi-implicit first order Euler scheme with only the linear highest gradient term being treated implicitly. We thus can compute the new material distribution *u*^*n*^ from the previous distribution *u*^*n*−1^ showing us the direction of maximal decline. As described above this gives us the direction of the biggest change in rigidities for small changes in shape. A more detailed description of the gradient flow, the finite element approximation and the implementation details are provided in the Appendices A and B, respectively, in the supplement.

## Numerical Results and Comparison to Plant Morphology

To derive that appearing cross-sectional shapes of plant stems or petioles play an important part in their mechanical behaviour, we will present three numerical experiments and a comparison to the cross-sectional shapes of the aforementioned load-bearing elements of the selected plants. As we are solely interested in the contribution of the cross-sectional shape of a plant axes to the twist-to-bend ratio, we assume a fixed ratio of bending modulus and shear modulus $E/G\approx 2.7$ for all numerical experiments, which for isotropic materials corresponds to a Poisson’s ratio $\nu \in [0.2,0.5]$, a value range, that is reasonable to assume for many plant axes^29^. Note again that we only compare the main load-bearing element of the respective cross-section of the selected plant with the cross-sections from our simulations. Detailed morphological-anatomical descriptions of the biological models plants are derived in Appendix C in the supplement.

### U-shapes

In a first experiment we consider the shape optimisation problem (Eq. (2)) with weighting factors ${\sigma }_{1}=1,\,{\sigma }_{2}=-\,1$ and ${\sigma }_{3}=0$. This corresponds to a minimisation of the torsional rigidity *D*_*z*_ and a maximisation of the minimal flexural rigidity *D*_min_. The small weighting factor for the perimeter regularisation is set to $\gamma \approx 1.4\cdot {10}^{-2}E$.

#### Description of the simulation

**Figure 2:**
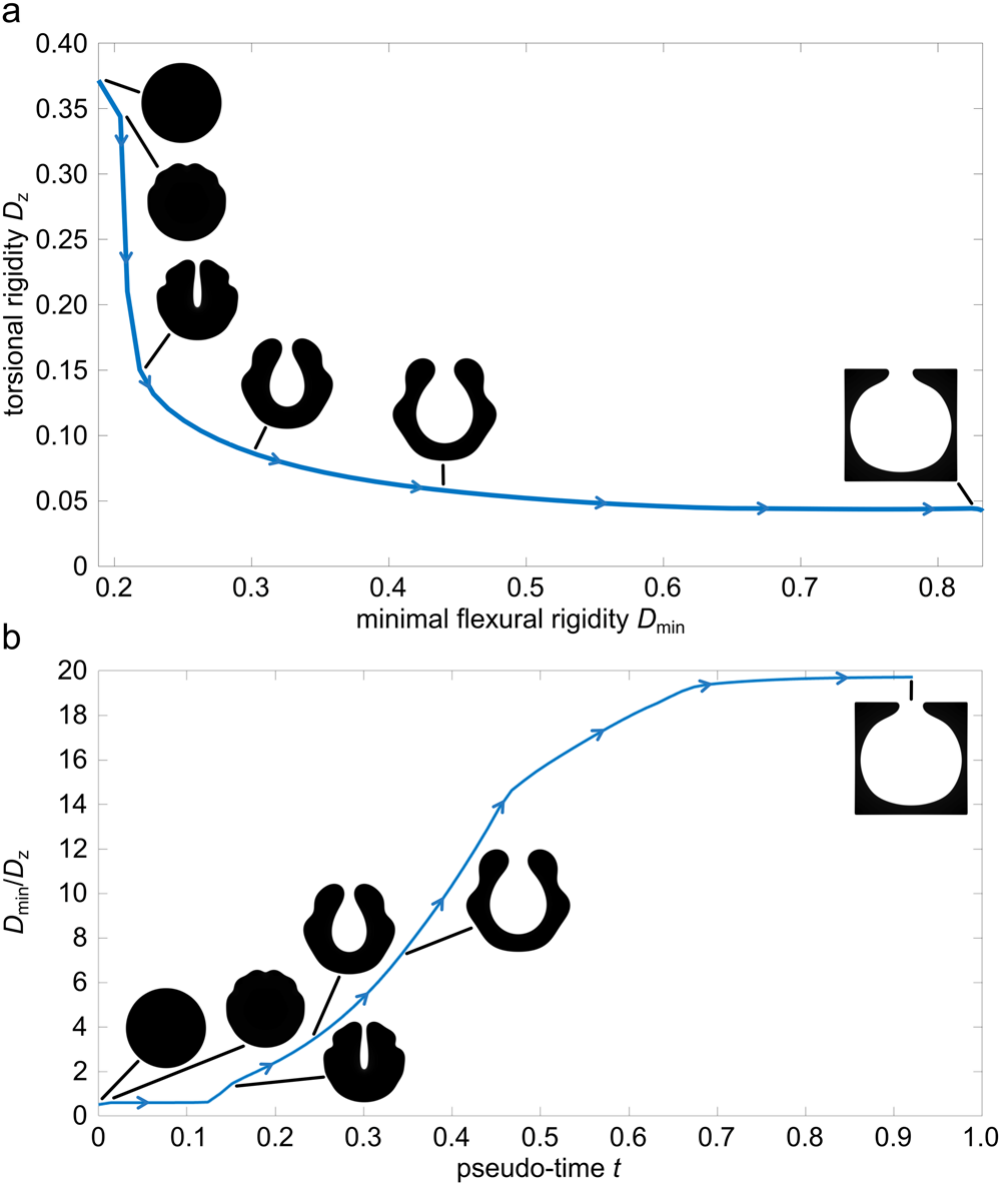
Evolution of the phase field in terms of maximising minimal flexural rigidity *D*_min_ and minimising torsional rigidity *D*_*z*_, (${\sigma }_{1}=-\,{\sigma }_{2}=1,{\sigma }_{3}=0$). (**a**) Evolution of the shape of the phase field with respect to torsional rigidity *D*_*z*_ (vertical axis) and flexural rigidity *D*_min_ (horizontal axis). (**b**) Evolution of the shape of the phase field with respect to the twist-to-bend ratio *D*_min_/*D*_*z*_ and pseudo-time *t*. The development of a deep central groove leads to a noticeable reduction in torsional rigidity, see (**a**) and thus to a first strong increase in the ratio, see (**b**). The flexural rigidity is then greatly increased by a widening of the central groove, (**a**) leading to another markedly increase in the twist-to-bend ratio. These two effects ultimately form the characteristic U-shaped domain. The numerical steady state is effected by the chosen artificial boundary conditions and is not considered in the comparison to plant morphology.

The evolution of the phase field is shown in Fig. 2. During the evolution, the circular initial shape of the plant petioles changes noticeably. After a short time period, small grooves form on the outer boundary of the phase. Such grooves are known to facilitate the twisting of a geometry as described by Vogel^7^. The results of the experiment confirm his finding and indicate that groove formation is the first dominant mode to reduce torsional rigidity starting from a circular disc as rod cross-section. The flexural rigidity barely changes in this initial phase.

During the further optimisation steps this trend becomes clearer (Fig. 2a). The central groove deepens further and leads to an even greater reduction in torsional rigidity, which results in a major increase in the twist-to-bend ratio (Fig. 2b). After half of the optimisation pseudo-time has passed, the flexural rigidity increases perceptibly for the first time. This effect can be attributed to the widening of the central groove, which causes the phase to shift outwards, building a U-shaped domain. This process continues until the phase has reached the boundary of the reference domain $\Omega$ ($t=0.35$). As soon as the phase contacts the boundary, the resulting shapes of course cannot be compared to plant morphology anymore. A numerical steady state of the gradient flow is obtained at pseudo-time $t\approx 0.8$.

#### Comparison with the leaf stalk of bananas (*Musa* sp.)

**Figure 3:**
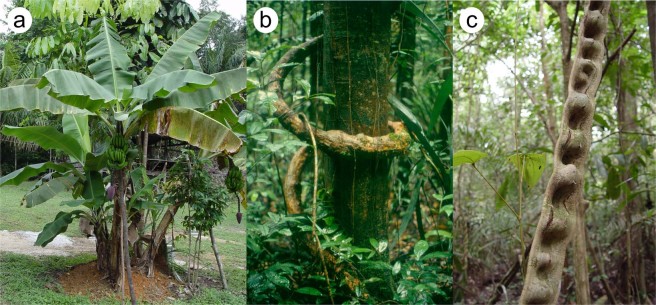
Selected model plants that were the basis for the comparative studies with the phase field simulations. (**a**) Banana plant of the species *Musa x paradisiaca* with its pseudostem composed of leaf sheaths and huge leaf laminae, (**b**) the stem of the twining liana *Condylocarpon guianense* wounding around a tree in the tropical rain forest of French Guyana, (**c**) a non-self-supporting ribbon-shaped stem of the liana *Bauhinia guianensis*, which is referred to as the monkey ladder because of its appearance growing in the tropical rain forest of French Guyana.

The shape change of the phase in this simulation shows great similarities with the cross-sections of banana leaf stalks (*Musa* sp.) (Fig. 3a and Fig. C1 in the supplement). Generally, leaf stalks (=petioles) should resist static loads such as bending caused by the leaf weight in order to hold the large leaf blade (=lamina) in place and to ensure its orientation towards the sun. Additionally, they have to withstand high dynamic loads caused, in particular, by wind forces acting on the lamina. These drag forces can be reduced by streamlining in the wind in terms of twisting the petiole^7,9,11,12,30,31^. This is extremely important for the integrity of the herbaceous banana plant, which consists of a pseudostem of densely packed leaf sheaths at the base of the petioles and leaf laminae with large surfaces, the latter being especially susceptible to damage from wind forces.

**Figure 4:**
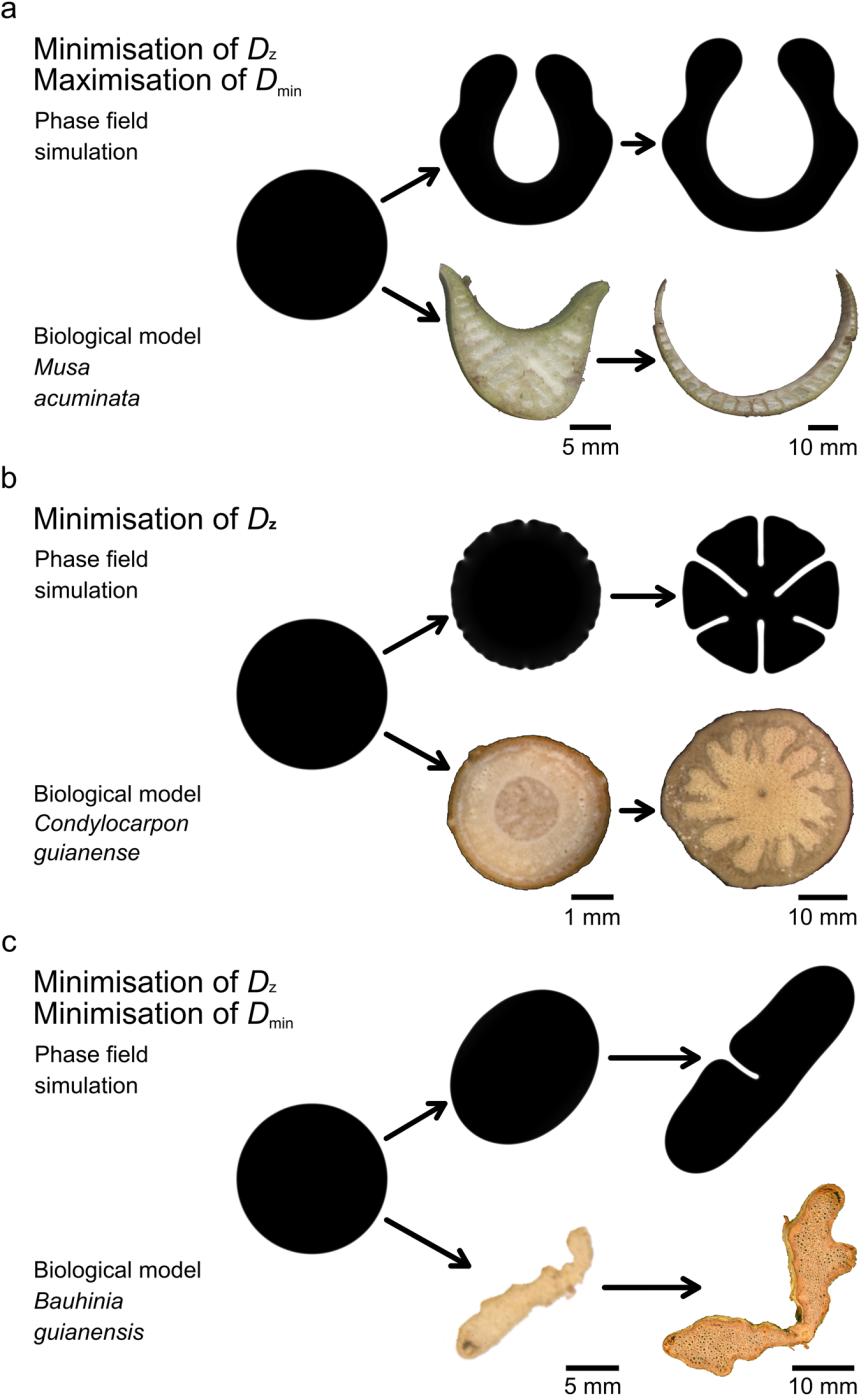
Comparison of individual shapes from the phase field simulations with cross-sections of the selected biological plant models. (**a**) Individual shapes of a phase field simulation with the aim of minimising the torsional rigidity *D*_*z*_ and of maximising the minimal flexural rigidity *D*_min_ are compared with cross-sections of the petiole of *Musa acuminata*. In *M*. *acuminata* the differences in the cross-sectional shape are attributable to the distinct positions along the longitudinal axis of the petiole (left = cross-section of the middle part, right = cross-section of the basal part). (**b**) Comparison between individual shapes of a phase field simulation in which only the torsional rigidity *D*_*z*_ is minimised and cross-sections of the stem of the liana *Condylocarpon guianense* in two different ontogenetic phases (left = self-supporting early stage, right = non-self-supporting old stage after attachment to a support). Reproduced from Rowe *et al*.^35^ with permission. (**c**) Individual shapes of a phase field simulation aimed at minimising the torsional rigidity *D*_*z*_ and minimising the minimal flexural rigidity *D*_min_ compared with cross-sections of a young lianescent (left) and an old lianescent stem (right) of the liana *Bauhinia guianensis*.

Morphological-anatomical studies of the U-shaped cross-section of banana petioles have revealed an inner and an outer shell comprising an epidermis and fibre-reinforced parenchyma with radial (sometimes branched) parenchymatous strands lying in between^11,32–34^ (Fig. 4a and Fig. C1 in  the supplement). This cross-sectional arrangement is associated with an increase in flexural rigidity, since its special structure prevents the petiole from bending downwards (and finally collapsing) by converting bending forces into tensile forces. In addition, the combination of shape and inner structure also reduces torsional rigidity and thus supports streamlining by torsion. These characteristics lead to extremely high twist-to-bend ratios of petioles of banana plants with values ranging from 40 to 100^9,11^, compared with petioles of various tree species with values between 1.6 and 9^7,12^. A comparable U-shape is visible in the phase field simulation alongside the trend towards an increasingly higher twist-to-bend ratio (Fig. 2).

The shape of the phases at various pseudo-times shows striking similarities with individual cross-sections along the longitudinal axis of the banana petiole^33,34^. The phase shapes at pseudo-times $t=0.3438$, $t=0.2475$ and $t=0.1512$ correspond well to the cross-sectional shapes found in the basal, middle and apical parts of the petioles (Fig. 2b, as well as Figs. C1 and C2 in the supplement). Figure 2b clearly shows that, with increasing pseudo-time, the torsional rigidity decreases, whereas the bending rigidity increases, and the resulting twist-to-bend ratio increases strongly in the relevant period between $t=0.1512$ and $t=0.3438$. From the viewpoint of functional morphology and biomechanics, this shape-dependent increase of the twist-to-bend ratio is strongly related to the mechanical loading of banana leaves. As a result of the increased leverage, because of the own weight of the leaf itself, the flexural rigidity in the basal part of the petiole has to be larger than that in the apical parts. For the torsional rigidity, on the other hand, it is advantageous to be uniformly low over the entire petiole in order to allow easy twisting under wind loads and thus to protect the leaf stalk from damage^11,31^.

### Deep grooves

In a second experiment we consider Eq. (2) with ${\sigma }_{2}={\sigma }_{3}=0$ and ${\sigma }_{1}=1$, which leads to a minimisation of torsional rigidity only. The weighting factor for the perimeter regularisation is $\gamma =1\cdot {10}^{-2}\,G$. We note that the bending modulus does not play a role here since the objective function does not include bending.

#### Description of the simulation

**Figure 5:**
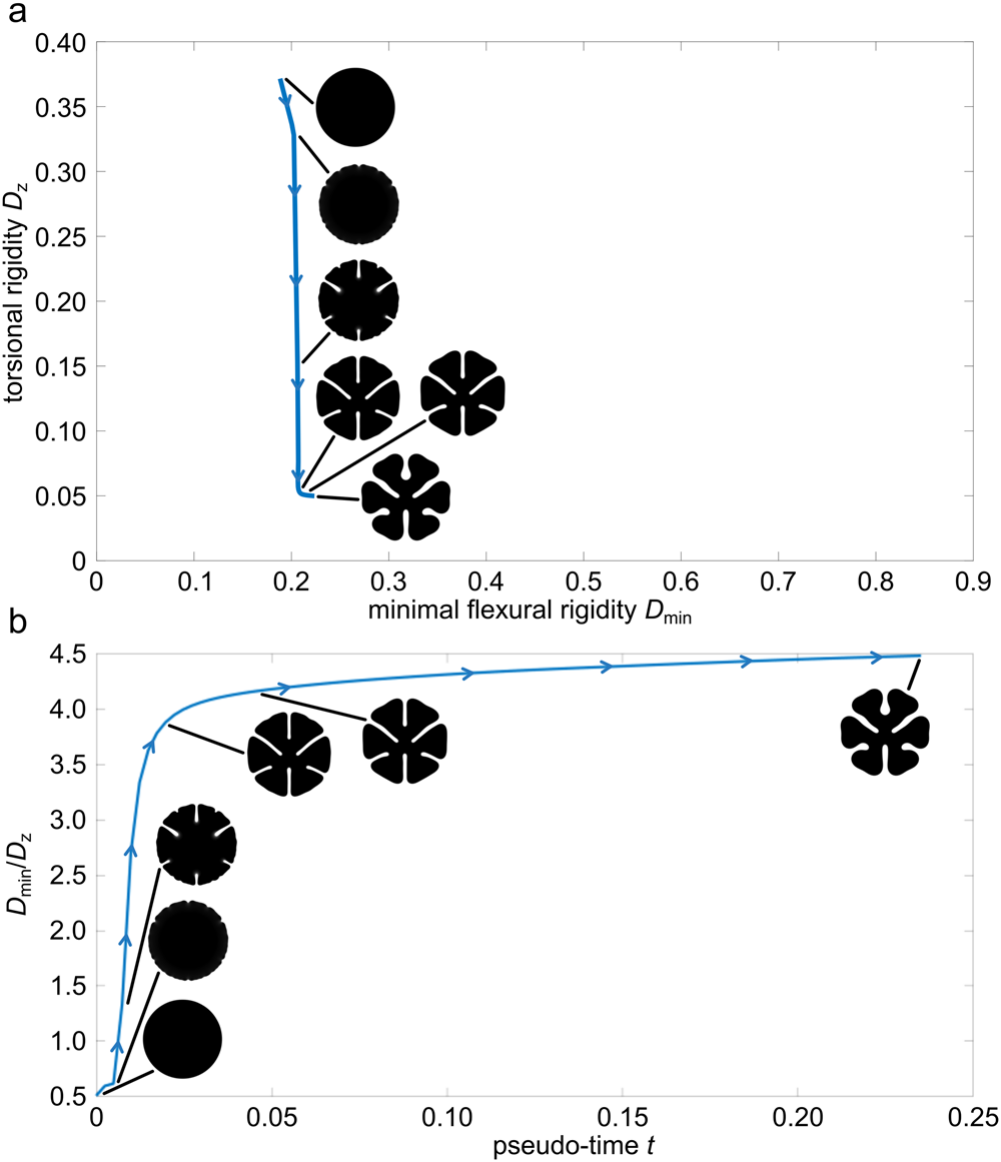
Evolution of the phase field in terms of minimising the torsional rigidity *D*_*z*_ only, (${\sigma }_{1}=1,{\sigma }_{2}={\sigma }_{3}=0$). (**a**) Evolution of the shape of the phase field with respect to torsional rigidity *D*_*z*_ and minimal flexural rigidity *D*_min_. (**b**) Evolution of the shape of the phase field with respect to the twist-to-bend ratio *D*_min_/*D*_*z*_ and pseudo-time *t*. Characteristic for the sole minimisation of the torsional rigidity is the developement of uniformly distributed deep grooves around the phase boundary resulting in a cloverleaf-shaped cross-section. Compared to the first experiment, a significant decrease in torsional rigidity occurs in a very short period of time, as seen in (**b**).

The evolution of the phase field is shown in Fig. 5. Similar to the first simulation, grooves appear again at the boundary of the phase field shape, which reduce the torsional rigidity. In contrast to the first experiment, however, these grooves appear uniformly distributed along the boundary (Fig. 5a).

The sole weighting of the torsional rigidity then leads to an even deepening of some grooves. By this, the twist-to-bend ratio is increased strongly after a comparatively short period of time and a characteristic cloverleaf-shaped cross-section is formed (Fig. 5b). A widening of the grooves, as in the experiment described above, only occurs to a lesser extent. The following smoothening of the phase boundary has no major influence on the torsional and flexural rigidity, respectively. A numerical steady state is reached at $t\approx 0.1435$. The flexural rigidity increases only marginally during the simulation and has no considerable influence on the twist-to-bend ratio. Compared to the first simulation the running pseudo-time needed to reach the steady state and thus the optimum under the given boundary conditions, is much lower.

#### Comparison with the stem of the liana *Condylocarpon guianense*

In this case, the shape change of the phase field has similarities with the ontogenetic developments of the cross-sectional shape of the woody part and thus the main load-bearing tissues of the stems of the liana *Condylocarpon guianense* (Fig. 3b). The rod-shaped stems of this species respond to the typical mechanical loads to which they are subjected in a certain ontogenetic phase by changes in their internal structure and in the material properties of the tissues involved. In young self-supporting *C*. *guianense* shoots, which are still searching for a support and are therefore mainly exposed to bending loads, a dense and stiff type of wood (secondary xylem) is formed. It is arranged in a centripetal pattern, with a central pith and an adjacent ring of dense wood (secondary xylem consisting of narrow-diameter vessels and small wood rays). This wood type is responsible for the relatively high flexural rigidity of young *C*. *guianense* stems, enabling them to bridge the distance to potential supports. As soon as the stems are securely attached to a support, a different type of wood is built, which is significantly less dense and mechanically more flexible (secondary xylem comprising wide-diameter vessels and broad wood rays forming grooves in the wood cylinder), contributing to the pronounced flexibility of old lianescent stems. Because of the formation of the two wood types in subsequent ontogenetic phases, young “searchers” form a dense and stiff wood cylinder, which is surrounded by lianoid non-dense wood built during older phases^16,35–38^ (Fig. 4b and Fig. C2 in the supplement).

Similar to the woody part of a young stem of *C*. *guianense*, the phase field simulation starts with a circular cross-section ($t=0.0000$) (Fig. 5 and Fig. D3 in the supplement). The twist-to-bend ratio of the phase field is very low at this point. However, within a very short period of time, especially compared with the first simulation, the twist-to-bend ratio of the simulated phase shape increases strongly (Fig. 5b). This increase in the twist-to-bend ratio can be attributed to the formation of the above mentioned grooves that strongly reduce the torsional rigidity while maintaining the flexural rigidity (Fig. 5a). Figuratively this can be imagined such that, because of the grooves, the largest possible resulting circular area of the cross-section is reduced, an event that is ultimately responsible for the torsional rigidity.

The resulting deeply grooved shape of the phase field is similar to the cross-sectional shape of the wood in older *C*. *guianense* stems. These older stages, which are by now attached to a support, are highly flexible in both bending and twisting and therefore allow the slender liana stem passively to follow the movement of the host tree under wind loading and even to survive the breakage of branches of even the entire stem of supporting host tree^35,36,39^ (Fig. 3b). The shape and three-dimensional arrangement of the wood within the older cross-sections differ markedly from those of the younger stages. Only a small circular ring of the dense wood remains around the central pith, while the lianoid less-dense wood is arranged in a deeply grooved (star-like) cross-sectional shape, analogous to the phase field, and fills most of the cross-sectional area^35^. We can thus assume that the decrease in torsional rigidity in older stages of *C*. *guianense* is (mainly) attributable to the different shape and arrangement of wood within the cross-sections of young and old stems.

**Figure 6:**
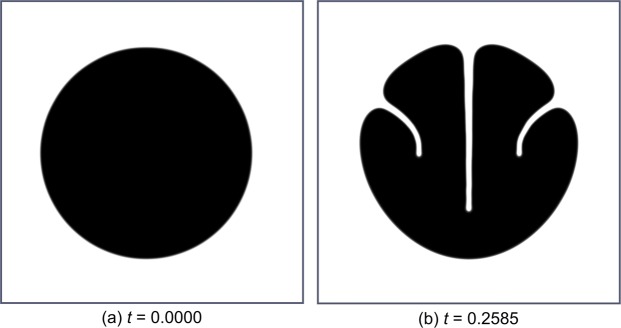
Phase field states of the simulation in terms of minimising the torsional rigidity *D*_*z*_ and additionally minimising the maximum flexural rigidity *D*_max_, (${\sigma }_{1}={\sigma }_{3}=1,{\sigma }_{2}=0$). (**a**) Initial state, (**b**) final state (with respect to pseudo-time *t*). Compared to the simulation shown in Fig. 5, merely minimising torsional rigidity, the additional minimisation of *D*_max_ results in a markedly different shape. This gives rise to the conjecture that the minimisation of the torsional rigidity is the main driving factor for the morphology of the main load bearing tissue in non-self-supporting old stages of *Condylocarpon guianense*.

If, in addition to the reduction of the torsional rigidity, a minimisation of the flexural rigidity is also included in the simulation, as is the case during the ontogeny of *C*. *guianense*, the resulting shape phase differs markedly from the real shape of the xylem in *C*. *guianense* stems (Fig. 6b). Consequently, we can reasonably assume that the decrease in flexural rigidity in *C*. *guianense* is primarily determined by the modified material properties of the wood formed in the lianescent phase of growth^16^ and not by the shape and 3D arrangement of the tissues involved.

In general, the self-supporting early stages of lianas show the typical values for flexural rigidity, as they are also known for other self-supporting woody stems. In contrast, the non-self-supporting older ontogenetic stages of lianas that are attached to a host support have considerably lower values (reduction of up to an order of magnitude). Although fewer data exist concerning torsional rigidity in the literature, the values of torsional rigidity of the secondary wood do not seem to decrease in the same way as the values of flexural rigidity^9^. To summarise, all lianas tested to date develop especially low ${D}_{{\rm{\min }}}/{D}_{z}$ ratios after “giving up” self-support^9^.

### Ribbons

In a third experiment we consider Eq. (2) with ${\sigma }_{3}=0$ and ${\sigma }_{1},{\sigma }_{2}=1$, which leads to a minimisation of both torsional rigidity and minimal flexural rigidity. The perimeter penalty is set to $\gamma \approx 1.4\cdot {10}^{-2}$, as in the first experiment.

#### Description of the simulation

**Figure 7:**
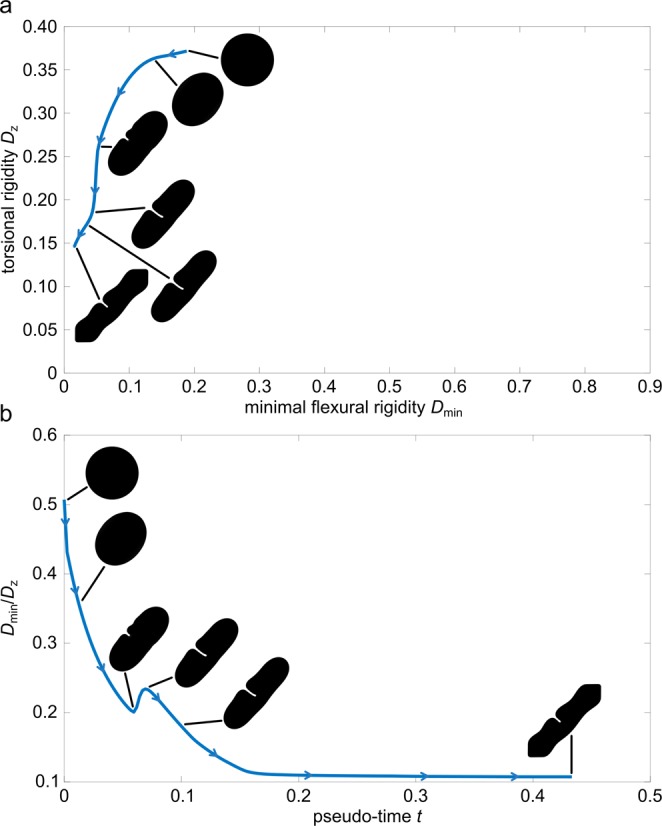
Evolution of the phase field in terms of minimising the torsional rigidity *D*_*z*_ and the minimal flexural rigidity *D*_min_ (${\sigma }_{1}={\sigma }_{2}=1,{\sigma }_{3}=0$). (**a**) Evolution of the shape of the phase field with respect to torsional rigidity *D*_*z*_ and flexural rigidity *D*_min_. (**b**) Evolution of the shape of the phase field with respect to the twist-to-bend ratio *D*_min_/*D*_*z*_ and pseudo-time *t*. Minimisation of both torsional and minimal flexural rigidity leads to ribbon-like domains. Compared to the previous experiments we now obtain one direction with low and another direction with high flexural rigidity, see (**b**) and Figs. D1 and D4 in the supplement.

The evolution of the phase field is shown in Fig. 7. A minimisation of both torsional and flexural rigidity allows the phase to form a nearly elliptic shape, leading to a noticeable decrease in both the torsional and the minimal flexural rigidity (in direction of the short axis), whereas the maximal flexural rigidity in the direction of the long axis is increased. A formation of grooves also occurs here. This is shown by a large groove in the middle of the shape, which like in the previous experiments deepens further, in this case leading to an even greater reduction in both torsional and minimal flexural rigidity. Before the phase touches the boundary the characteristic shape appears at $t=0.1040$. A numerical steady state is reached at pseudo-time $t\approx 0.3960$. For more details see Fig. D4 in the supplement.

Considering the twist-to-bend ratio of the shapes, it is important to say that contrary to the previous experiments, only the ratio ${D}_{{\rm{\max }}}/{D}_{z}$ is markedly increased (Fig. D1 in the supplement), where the ratio ${D}_{{\rm{\min }}}/{D}_{z}$ is reduced (Fig. 7b). The cross-sectional shapes that occur thus only show a high twist-to-bend ratio in the direction of the maximum flexural rigidity. In the previous experiments these two ratios were almost identical due to the near symmetry of the cross-sectional shapes.

**Figure 8:**
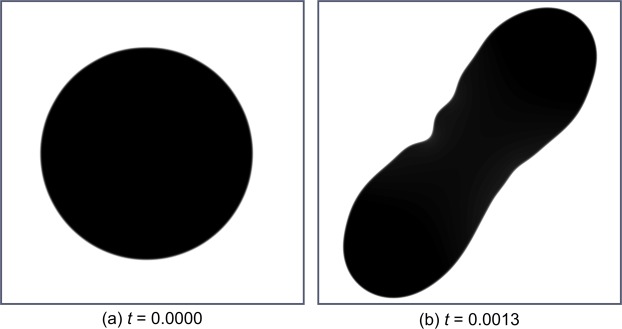
Phase field states of the simulation minimising the torsional rigidity *D*_*z*_ as well as minimum flexural rigidity *D*_min_ and additionally maximising maximum flexural rigidity *D*_max_, (${\sigma }_{1}={\sigma }_{2}=1,{\sigma }_{3}=-\,1$), (**a**) Initial state, (**b**) final state (with respect to pseudo-time *t*). Compared to the simulation shown in Fig. 7, the additional maximisation of *D*_max_ prevents the developement of the deep groove in the centre.

The mentioned groove in the middle of the shape is the decisive characteristic, which distinguishes this model from the model with an additional maximisation of the maximum flexural rigidity *D*_max_. The formation of such a groove slows the increase in maximum flexural rigidity *D*_max_ and is thus suppressed when the maximum flexural rigidity is included as an objective to be maximised (Fig. 8).

#### Comparison with the stem of the monkey ladder liana (*Bauhinia guianense*)

Like the stems of *Condylocarpon guianense*, the stems of the liana *Bauhinia guianensis* change their mechanical properties, wood type and wood shape markedly during ontogeny^16^. Young self-supporting stems and young apical axes of *B*. *guianensis* have a circular cross-sectional shape composed of a central pith and an adjacent ring of dense stiff wood (secondary xylem consisting of narrow-diameter vessels and small wood rays) (Fig. 4c and Fig. C3 in the supplement). These young axes are stiff in both bending and torsion^16,37,39^. As in *C*. *guianense* the young shoots act as “searchers”, spanning gaps between the host supports (Fig. 3c) and therefore rely on high values of flexural and torsional rigidity^36,37,39^.

Similar to the cross-sections of young *B*. *guianensis* stems, the phase field simulation starts from a round shape ($t=0.00$) (Fig. 4c and Fig. D4 in the supplement), which also features high minimal flexural rigidity and high torsional rigidity and thus the highest twist-to-bend ratio within this simulation (Fig. 7). In contrast, adult non-self-supporting lianescent stems of *B*. *guianensis* are much more flexible and have a markedly lower modulus of elasticity and their cross-sectional shape differs considerably from that of young stems^16^. These changes in the mechanical properties of the stem have been correlated with changes in the contribution of the various wood types (small amounts of dense stiff secondary xylem built in the young self-supporting stage and large amounts of non-dense flexible secondary xylem with wide-diameter vessels and broad wood rays formed after attachment in the lianescent stage) to the axial second moment of area of the stems^16,37^. This also becomes apparent with regard to the change in the cross-section of the stem from a circular to a ribbon shape during the ontogeny of the plant^39^. The ribbon shape, which gives *B*. *guianensis* its vernacular name of “monkey ladder”, can also be seen in the phase field simulation and is associated with reductions in minimal flexural rigidity, torsional rigidity and twist-to-bend ratio. Moreover, as described above, the phase field shape exhibits a mid-line groove, which further decreases the minimal flexural rigidity and the torsional rigidity. This groove is also present in *B*. *guianensis* but has a slightly different shape. In the actual plant, the groove is much wider towards the outside than the groove in the phase field simulation.

Regardless of their individual shape, grooves have a similar effect on the mechanics of the overall structure. Figuratively speaking, the grooves reduce the largest possible resulting circular area within the cross-section, which ultimately leads to a decrease of the torsional rigidity. Another similarity with the phase field simulation is the cross-sectional stem shape of *B*. *guianensis* in the transition phase from young to adult stages. Since additional large-lumen wood is only formed on two opposite sides of the young circular stem, the cross-section shows more and more similarities with the elliptical shape of the phase field shortly after the start of the simulation (Fig. 4c). The simulation reveals that this change in the cross-sectional shape results in a simultaneous decrease of the torsional rigidity and of the minimal flexural rigidity (Fig. 7a).

## Discussion

In summary, some similarities, but also some differences, exist between the three experiments, namely the simulations of “U-shapes”, “Deep grooves” and “Ribbons”. The simulations of “U-shapes” and “Deep grooves” are readily comparable insofar that, dependent on the various weighting factors, both lead to an increase of the twist-to-bend ratio. Comparison of these two simulations demonstrates clear differences with regard to the increase and the maximum values of the twist-to-bend ratio. With the exclusive minimisation of torsional rigidity, as performed in “Deep grooves”, a twist-to-bend ratio of ${D}_{{\rm{\min }}}/{D}_{z}\approx 4$ can be achieved even after a pseudo-time of $t\approx 0.025$, whereas with the minimisation of the torsional rigidity and a simultaneous maximisation of the minimum flexural rigidity, as was carried out in “U-shapes”, a twist-to-bend ratio of ${D}_{{\rm{\min }}}/{D}_{z}\approx 4$ could only be reached after a pseudo-time of $t\approx 0.26$. On the other hand, the overall twist-to-bend ratio is higher if the minimal flexural rigidity is additionally maximised, as conducted in “U-shapes”, with twist-to-bend ratios of ${D}_{{\rm{\min }}}/{D}_{z}\approx 20$, instead of just the minimisation of the torsional rigidity as performed in “Deep grooves”, where the maximal twist-to-bend ratio only has values of ${D}_{{\rm{\min }}}/{D}_{z}\approx 4.5$. Interestingly, in the simulation of “Ribbons”, the twist-to-bend ratio ${D}_{{\rm{\min }}}/{D}_{z}$ decreases over time, whereby the ${D}_{{\rm{\max }}}/{D}_{z}$ increases and achieves after a pseudo-time of $t\approx 0.1$, a twist-to-bend ratio of ${D}_{{\rm{\max }}}/{D}_{z}\approx 4$ and maximum values of ${D}_{{\rm{\max }}}/{D}_{z}\approx 5.5$.

Apart from these differences, a common shape-related characteristic noticeably occurs in all three simulation, namely the formation of grooves. Figuratively, these grooves reduce the largest possible circular area that can be placed in the phase field shapes, whose size corresponds to the torsional rigidity and thus ultimately leads to a reduction in the torsional rigidity of the overall structure. Since all simulations are at least partly aimed at minimising the torsional rigidity, we can expect that these grooves will occur in all three simulations. Only the design of these grooves varies depending on the additional optimisation requirements.

What conclusions can be drawn from these findings for the selected plant models? Since plants as biological structures are generally the result of multifunctional requirements and, moreover, can only respond or adapt within the framework of their respective bauplan, the influence of the shape of a structure on the overall performance in terms of flexural and torsional rigidity cannot be derived from the plant models. With the simulations presented here, this assignment is possible for the first time, although the twist-to-bend ratio is clearly a measure for a compromise of various mechanical functions. Possible deviations of plant axes from the optimised shape are indications for further functions that are vital for the survival of the respective plant species. Precisely for this reason and because the twist-to-bend ratio is a dimensionless parameter, it is particularly suitable for comparing biological structures not only with each other, but also with technical structures.

Experimental investigations on the petiole of the banana leaf have shown a twist-to-bend ratio ranging from 40 to 100^11,32^. Analogous to the various phase field shapes found in the simulation of “U-shapes”, the banana petiole also displays various cross-sectional shapes along its longitudinal axis and thus a change in mechanical functionality. In addition to this spatial resolution based on the various cross-sectional shapes, a difference exists between the theoretically achievable maximum value of ${D}_{{\rm{\min }}}/{D}_{z}\approx 20$, as determined in the simulation “U-shapes” and purely resulting from the respective shape, and the values determined experimentally. This difference can only be explained by the special inner structure of the petiole. The fact that the banana petiole is up to 100 times stiffer in bending than in torsion represents a selective advantage with regard to the alignment of the leaf blade to sunlight in the sense of efficient photosynthesis and simultaneously avoids damage to the leaf blade, as the leaves are streamlined under wind load.

In contrast to the banana leaf, which represents a spatial resolution of various cross-sectional shapes, the two selected liana species have a temporal resolution of the different cross-sectional shapes as a function of ontogenetic development from the young and old ontogenetic stages. First of all, the various stages differ mainly in their mechanical properties: young stages are self-supporting and are stiff “searchers”, whereas the older stages are safely attached to the host support and are non-self-supporting and characterised by high flexural and torsional flexibility. The reduction in flexural and torsional rigidity takes place via rapid transitions from dense stiff wood built in the early stages to less-dense flexible wood developed in the older stages. Later shifts in development include the change in the cross-sectional shape by the formation of woody lobes and resulting grooves as described in simulations “Deep grooves” and “Ribbons”.

Specifically, the bending modulus *E* of *C*. *guianense* axes decreases from a mean of 2722 MPa during early ontogenetic stages to a mean of 306 MPa during older stages, whereas the percentage contribution of the wide-lumen wood to the cross-sectional area increases from 0 to 30%^16,35,36^. From the simulation of “Deep grooves”, we learn that the minimisation of the torsional rigidity of *C*. *guianense* axes with almost constant bending rigidity is controlled by the increasing lobation of the cross-sectional shape of the wood. We can conclude from this observation that additional flexural flexibility is controlled by the formation of flexible lianoid wood having other material properties.

Similar to *C*. *guianense*, changes in the cross-sectional shape and mechanical behaviour of *B*. *guianensis* stems are linked to the ontogenetic stage of the plant. Early stages with a circular cross-section producing dense stiff wood are 2–3 metres long and occur as self-supporting “searchers” that can bridge the gap to potential host supports or self-supporting young saplings^39^. As soon as the stem is attached to a supporting tree, rapid transitions to compliant wood take place. Interestingly, the cambial growth is highly modified producing a ribbon-shaped stem attributable to the formation of lianoid wood at two opposite sides of the young circular stem and changes into an elliptical cross-section^39^. During the period between wood built in young stages to wood built in older stages, the bending modulus *E* decreases from 24 GPa to 3.75 GPa and the torsional modulus *G* decreases from 0.91 GPa to 0.42 GPa^16^. The simulation of “Ribbons” shows that the torsional flexibility at almost constant minimal flexural rigidity (${D}_{{\rm{\min }}}/{D}_{z}$) is controlled by the shape change from circular to elliptical and the additional formation of one groove at the centre of the major axis. This above-mentioned rapid transition from one stage to the other is mirrored in the phase field simulation of “Ribbons” in which a relatively short pseudo-time is required to optimise the twist-to-bend ratio ($t\approx 0.2$) for reaching the lowest value ${D}_{{\rm{\min }}}/{D}_{z}\approx 0.1$. This is different when the twist-to-bend ratio ${D}_{{\rm{\max }}}/{D}_{z}$ is considered. Here, the flexural rigidity can be 6 times as high as the torsional rigidity.

## Conclusion

The use of gradient flow functions in the form of phase field simulations has proved to be a novel and appropriate approach that helps us to understand optimisation processes during evolution and ontogeny within biology. In the framework of this study, the gradient flow has been used to illustrate the fastest/largest possible changes in rigidity with the smallest possible change in the cross-sectional shape of the load-bearing structures. A comparison with selected plant species suggests that evolution also follows this principle, as small changes in cross-sectional shape are “easy to implement” at little “costs”, but still offer a large selective advantage. This approach can probably also be used to aid our understanding of other evolutionary or ontogenetic optimisation processes.

## Supplementary information

Supplement 1 – Appendices

Supplement 2 – Numerical Program Code

## Data Availability

This work does not have any experimental data. The shape-optimisation C++-code is made available as supplementary material.
