# Supplementary material for: Twist-to-bend ratio: an important selective factor for many rod-shaped biological structures
Source: Sci Rep. 2019 Nov 20;9:17182. doi: 10.1038/s41598-019-52878-z (PMC6868162; doi:10.1038/s41598-019-52878-z)
Supplement: Supplementary file 1 — Supplement 1 – Appendices [file 41598_2019_52878_MOESM1_ESM.pdf]

Twist-to-bend ratio: an important selective factor for many rod-shaped biological structures (Appendices)

Steve Wolff-Vorbeck[1], Max Langer[2], Olga Speck[2,3], Thomas Speck[2,3,4], and Patrick Dondl[1,*]

[1]Department of Applied Mathematics, University of Freiburg, Hermann-Herder-Str. 10, D-79104 Freiburg, Germany
[2]Plant Biomechanics Group, Botanic Garden, Faculty of Biology, University of Freiburg, Schänzlestraße 1, D-79104 Freiburg, Germany
[3]Cluster of Excellence *liv*MatS @ FIT – Freiburg Center for Interactive Materials and Bioinspired Technologies, University of Freiburg, Georges-Köhler-Allee 105, D-79110 Freiburg, Germany
[4]Freiburg Materials Research Center (FMF), University of Freiburg, Stefan-Meier-Str. 21, D-79104 Freiburg, Germany
[*]patrick.dondl@mathematik.uni-freiburg.de

Appendix A: L^2-Gradient Flow

Introducing an artificial time variable t (hereafter called pseudo-time) and using the L^2 scalar product $(\cdot,\cdot)$ as well as the first variation $\frac{\delta I}{\delta u}[v]$ of a functional I in u in the direction of v the choice of a gradient flow dynamic leads to the pseudo-time stepping approach given by

$$\varepsilon(\partial_t u, v)_{L^2(\Omega)} = -\frac{\delta I_\varepsilon}{\delta u}[v] = -\left(\sigma_1 \frac{\delta D_z}{\delta u}[v] + \sigma_2 \frac{\delta D_{\min}}{\delta u}[v] + \sigma_3 \frac{\delta D_{\max}}{\delta u}[v] + \gamma \frac{\delta \operatorname{Per}_\varepsilon}{\delta u}[v]\right) \tag{A1}$$

for all $v \in H_0^1(\Omega)$. The variation of the perimeter term yields the well-known Allen-Cahn equation. To solve equation (A1) by a finite element method, we thus still need to compute the first variations of $D_{\min/\max}(u)$ and $D_z(u)$ in u. Concerning $D_{\min/\max}(u)$ these are given by simple calculations and with

$$\frac{\delta D_x}{\delta u}[v] = E\left(\int_\Omega \hat{x}^2 v \, \mathrm{d}x\mathrm{d}y - 2\int_\Omega u b_1 \hat{x} \, \mathrm{d}x\mathrm{d}y\right),$$

$$\frac{\delta D_y}{\delta u}[v] = E\left(\int_\Omega \hat{y}^2 v \, \mathrm{d}x\mathrm{d}y - 2\int_\Omega u b_2 \hat{y} \, \mathrm{d}x\mathrm{d}y\right),$$

$$\frac{\delta D_{xy}}{\delta u}[v] = E\left(\int_\Omega \hat{y}\hat{x} v \, \mathrm{d}x\mathrm{d}y - \int_\Omega u(b_1\hat{x} + b_2\hat{y}) \, \mathrm{d}x\mathrm{d}y\right)$$

and $b_1 = \frac{1}{m}\int xv \, \mathrm{d}x\mathrm{d}y$, $b_2 = \frac{1}{m}\int yv \, \mathrm{d}x\mathrm{d}y$, we obtain

$$\frac{\delta D_{\text{mean}}}{\delta u}[v] = \frac{1}{2}\left(\frac{\delta D_x}{\delta u}[v] + \frac{\delta D_y}{\delta u}[v]\right),$$

$$\frac{\delta RM}{\delta u}[v] = \frac{(D_x - D_y)\left(\frac{\delta D_x}{\delta u}[v] - \frac{\delta D_y}{\delta u}[v]\right) + 4D_{xy}\frac{\delta D_{xy}}{\delta u}[v]}{2\sqrt{(D_x - D_y)^2 + 4D_{xy}^2}}.$$

For regularisation near $RM(u) = 0$, we introduce a parameter $0 < \theta_1 \ll 1$ and approximate $\frac{\delta RM}{\delta u}[v]$ by

$$\frac{\delta RM_{\theta_1}}{\delta u}[v] = \frac{(D_x - D_y)\left(\frac{\delta D_x}{\delta u}[v] - \frac{\delta D_y}{\delta u}[v]\right) + 4D_{xy}\frac{\delta D_{xy}}{\delta u}[v]}{2\sqrt{(D_x - D_y)^2 + 4D_{xy}^2 + \theta_1}}.$$

Finally, using $D_{\min/\max} = D_{\text{mean}} \mp RM$, the variation of the flexural rigidities can be computed.
Concerning the first variation of D_z, we consider the weak formulation of equation (4) which is given by

$$\int_\Omega \frac{1}{G(u+\theta_0)^2} \nabla\phi \cdot \nabla v \, \mathrm{d}x\mathrm{d}y = 2 \int_\Omega v \, \mathrm{d}x\mathrm{d}y \tag{A2}$$

for all test functions $v \in H_0^1(\Omega)$ and $\phi = 0$ on $\partial\Omega$.
Remark. We note that for every admissible phase field variable $u \in \mathscr{A}$ the Riesz representation theorem ensures that equation (A2) is uniquely solvable.
Since the variation of $D_z(u)$ depends on the solution $\phi(u)$ of equation (A2), we apply a Lagrangian approach, see, e.g., Hinze et al.[1]. Introducing the adjoint variable $p : \Omega \to \mathbb{R}$ we can formulate the Lagrangian as

$$L(u,\phi,p) = \mathrm{Per}_\varepsilon(u) + 2\int_\Omega \phi \, \mathrm{d}x\mathrm{d}y - \frac{1}{G}\int_\Omega \frac{1}{(u+\theta_0)^2} \nabla\phi \cdot \nabla p \, \mathrm{d}x\mathrm{d}y + 2\int_\Omega p \, \mathrm{d}x\mathrm{d}y.$$

Seeking stationary states (u,ϕ,p) of L, we find that, if the first variation for (u,ϕ,p) vanishes, both ϕ and p solve equation (A2). Since equation (A2) is uniquely solvable we conclude that $\phi = p$ and obtain

$$\frac{\delta L}{\delta u}[v] = \frac{\delta\,\mathrm{Per}_\varepsilon}{\delta u}[v] + \frac{1}{G}\int_\Omega \frac{2}{(u+\theta_0)^3} \nabla\phi \cdot \nabla\phi \cdot v \, \mathrm{d}x\mathrm{d}y$$

with the solution $\phi(u)$ of equation (A2). This finally gives us the gradient flow

$$\varepsilon(\partial_t u, v)_{L^2(\Omega)} = -\left(\sigma_1 \frac{\delta D_z}{\delta u}[v] + \sigma_2 \frac{\delta D_{\min}}{\delta u}[v] + \sigma_3 \frac{\delta D_{\max}}{\delta u}[v] + \gamma \frac{\delta\,\mathrm{Per}_\varepsilon}{\delta u}[v]\right)$$

with

$$\frac{\delta D_z}{\delta u}[v] = \frac{1}{G}\int_\Omega \frac{2}{(u+\theta_0)^3} (\nabla\phi \cdot \nabla\phi) v \, \mathrm{d}x\mathrm{d}y.$$

Appendix B: Finite Element Approximation

To solve equation (A1) by the finite element method we choose the discrete subspace $\mathscr{S}^{1,0}(\mathscr{T}_h) \subset H_0^1(\Omega)$, which is given by

$$\mathscr{S}^{1,0}(\mathscr{T}_h) := \{v_h \in C^0(\bar{\Omega})\colon (v_h)_{|T} \in P^1(T), \text{ for all } T \in \mathscr{T}_h\colon v_h = 0 \text{ on } \partial\Omega\}.$$

Further we choose a finite difference quotient in order to discretise the time derivative. Using an explicit treatment of the appearing nonlinear terms in equation (A1), and an implicit treatment of the Laplacian, we obtain the semi-implicit time stepping

$$\begin{aligned}
&\int_\Omega \varepsilon \frac{\mathscr{I}_{1,h}(u^{n+1}-u^n)}{\tau}\phi_j \,\mathrm{d}x\mathrm{d}y = -\int_\Omega \varepsilon\gamma\nabla\mathscr{I}_{1,h}(u^{n+1})\cdot\nabla\phi_j + \frac{\gamma}{\varepsilon}f(\mathscr{I}_{1,h}(u^n))\phi_j \,\mathrm{d}x\mathrm{d}y \\
&+\sigma_2\frac{\delta D_{\min}(\mathscr{I}_{1,h}(u^n))}{\delta\phi_j} + \sigma_3\frac{\delta D_{\max}(\mathscr{I}_{1,h}(u^n))}{\delta\phi_j} \\
&-\sigma_1\int_\Omega \frac{2}{(\mathscr{I}_{1,h}(u^n)+\theta_0)^3}\nabla\phi(\mathscr{I}_{1,h}(u^n))\cdot\nabla\phi(\mathscr{I}_{1,h}(u^n))\phi_j \,\mathrm{d}x\mathrm{d}y,
\end{aligned}$$

with inner nodal basis points $x_i \in \mathscr{N}_h$ and $j = 1,\ldots,|\mathscr{N}_h|$. The nodal interpolant $\mathscr{I}_{1,h}(v)$ of a function $v \in C^0(\bar{\Omega})$ is given by

$$\mathscr{I}_{1,h}v = \sum_{j\in\mathscr{N}_h}\phi_j v(x_j).$$

The mass constraint is imposed by the additional condition

$$\int_\Omega \mathscr{I}_{1,h}(u^n)\,\mathrm{d}x\mathrm{d}y - m = 0.$$

In every time step t we thus solve the problem

$$\begin{pmatrix} S & B \\ B^T & 0 \end{pmatrix}\cdot U^{n+1} = \tilde{F},$$

where $S = M + \tau\gamma K \in \mathbb{R}^{n\times n}$ and $B = L$. Here the mass matrix M, stiffness matrix K and lumped mass matrix $L \in \mathbb{R}^n$ (represented only by its diagonal) are given by

$$M_{ij} = \int_\Omega \nabla\phi_j\phi_i\,\mathrm{d}x\mathrm{d}y,\;\; K_{ij} = \int_\Omega \nabla\phi_j\cdot\nabla\phi_i\,\mathrm{d}x\mathrm{d}y,\;\; L_i = \int_\Omega \phi_i\,\mathrm{d}x\mathrm{d}y.$$

The right hand side $\tilde{F}$ is given by $\tilde{F}_j = \frac{\tau}{\varepsilon}F_j + (M\cdot U^n)_j$ for $j = 1,\ldots,N$, and $\tilde{F}_{N+1} = m$, where we have

$$\begin{aligned}
F_j = &\frac{\gamma}{\varepsilon}\int_\Omega f(\mathscr{I}_{1,h}(u^n))\phi_j\,\mathrm{d}x\mathrm{d}y \\
&+\sigma_2\frac{\delta D_{\min}(\mathscr{I}_{1,h}(u^n))}{\delta u^n}[\phi_j] + \sigma_3\frac{\delta D_{\max}(\mathscr{I}_{1,h}(u^n))}{\delta u^n}[\phi_j] \\
&-\sigma_1\int_\Omega \frac{2}{(\mathscr{I}_{1,h}(u^n)+\theta_0)^3}\nabla\phi(\mathscr{I}_{1,h}(u^n))\cdot\nabla\phi(\mathscr{I}_{1,h}(u^n))\phi_j\,\mathrm{d}x\mathrm{d}y.
\end{aligned}$$

In order to achieve an energy stability such that for all n we have

$$(\gamma\mathrm{Per}_\varepsilon + \sigma_1 D_z + \sigma_2 D_{\min} + \sigma_3 D_{\max})(u^n) \leq (\gamma\mathrm{Per}_\varepsilon + \sigma_1 D_z + \sigma_2 D_{\min} + \sigma_3 D_{\max})(u^{n-1}),$$

it is necessary to demand $\tau \in \mathscr{O}(\varepsilon^3)$.

For the experiments we set the interfacial parameter $\varepsilon = 3\cdot 10^{-3}$ and choose the time step as $\tau = 5.5\cdot 10^{-6}$. The parameters for the material elastic moduli are chosen to be $E = 71$, $G = 26$. For the domain Ω we use a triangulation $\mathscr{T}$ of the square $\Omega = [-1,1]^2$ with mesh size $h_{\mathscr{T}} \sim 10^{-3}$. The parameter θ_0 from equation (4) is set to $\theta_0 = \varepsilon$.

Appendix C: Model plants

Different cross-sections of the three model plants (*Musa acuminata*, *Condylocarpon guianense*, *Bauhinia guianensis*) with labelling of the mechanically important tissues. The three cross-sections of *Musa acuminata* represent different parts of a leaf petiole (Figure C1). For *Condylocarpon guianense* (Figure C2) and *Bauhinia guianensis* (Figure C3) cross-sections of the stems originate from different ontogenetic stages of each plant.

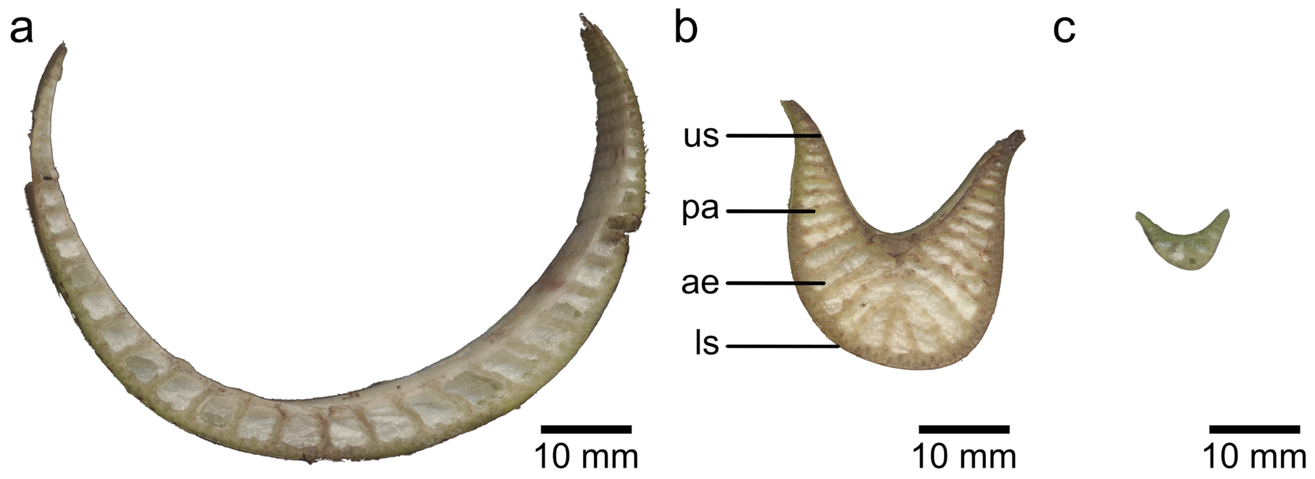

Figure C1. U-shaped cross-sections through various parts of a leaf stalk of *Musa acuminata*. Internally, the petiole consists of air-filled aerenchyma (ae) and parenchymatic strands (pa) that connect the upper shell (us) with the lower shell (ls). Section through (**a**) the basal part, (**b**) the middle part and (**c**) the apical part of the leaf stalk.

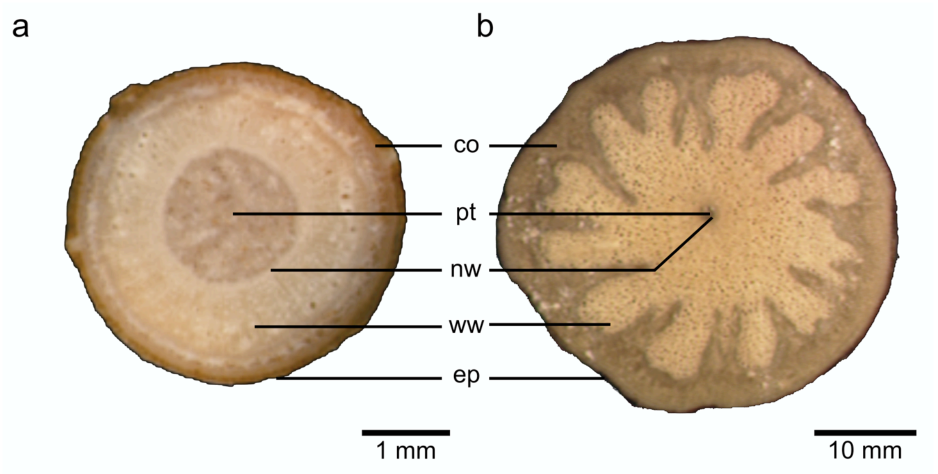

Figure C2. Cross-sections of *Condylocarpon guianense* stems at various ontogenetic stages. In general, both cross-sections show a central pith (pt), an adjacent ring of dense wood consisting of narrow-diameter vessels and small wood rays (nw), surrounded by less-dense wood comprising wide-diameter vessels and broad wood rays (ww), which itself is enclosed by cortex tissue (co) and the epidermis (ep). (**a**) The young stage exhibits a centripetal pattern of ring-shaped tissue layers around the central pith. (**b**) In the older stage, the central cylinder of stiff xylem represents the remnants of the young stage. In addition, a large amount of newly formed less-dense flexible xylem has formed showing a lobed cross-section with grooves of various depths. Reproduced from Rowe et al.[2] with permission.

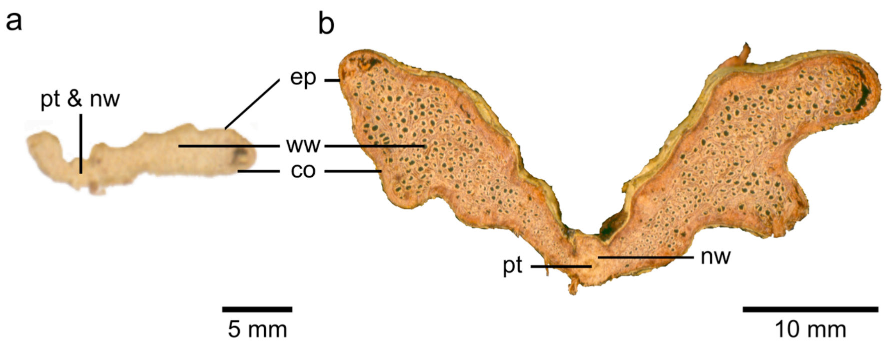

Figure C3. Cross-sections of *Bauhinia guianensis* stems at various ontogenetic stages. (**a**) The cross-section of a stem in the transition phase from an early to an older lianescent stage, comprising a central pith (pt) and an adjacent ring of dense wood consisting of narrow-diameter vessels and small wood rays (nw), surrounded by cortex tissue (co) and the epidermis (ep). (**b**) Cross-section of an adult stem in which a remnant from the young stage can be found in the centre. In addition, a huge amount of less-dense wood comprising wide-diameter vessels and broad wood rays (ww) only forms on two opposite sides of the young circular stem resulting in an elliptical shape with a wide groove.

Appendix D: Pseudo-time evolution of the different phase field simulations

We show the different shapes of the phase field at selected time steps. The evolution with respect to pseudo-time t of the gradient flow for minimising torsional rigidity and maximising minimum flexural rigidity $D_{\min}$ is displayed in Figure D2.

The evolution of the gradient flow for minimising merely torsional rigidity D_z is displayed in Figure D3.

The evolution of the gradient flow for minimising torsional and minimal flexural rigidity is shown in Figure D4. The same evolution along the graph of the twist-to-bend ratio is depicted in Figure D1.

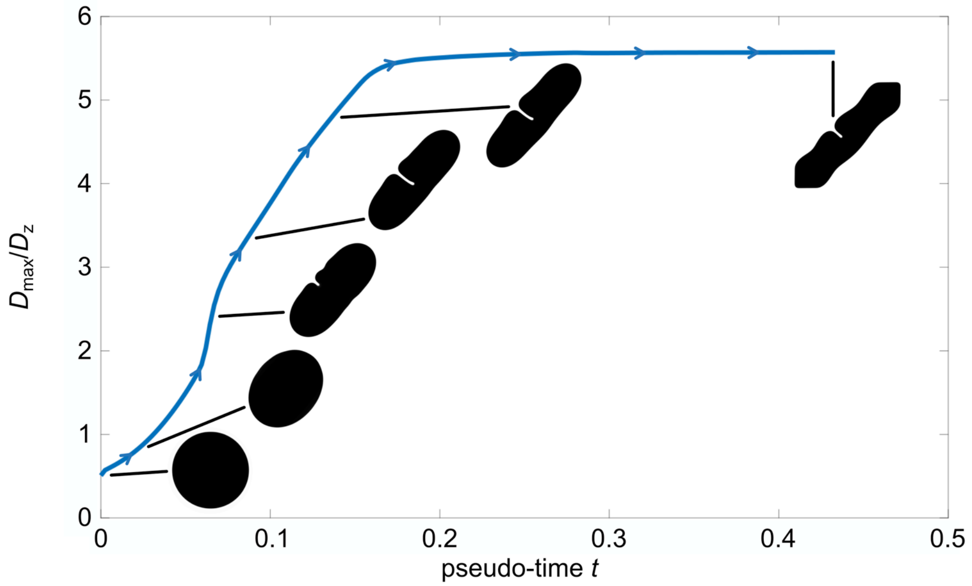

Figure D1. Evolution of the shape of the phase field in terms of minimising torsional rigidity and minimal flexural rigidity ($\sigma_1 = \sigma_2 = 1, \sigma_3 = 0$) with respect to the twist-to-bend ratio $D_{\max}/D_z$ and pseudo-time t.

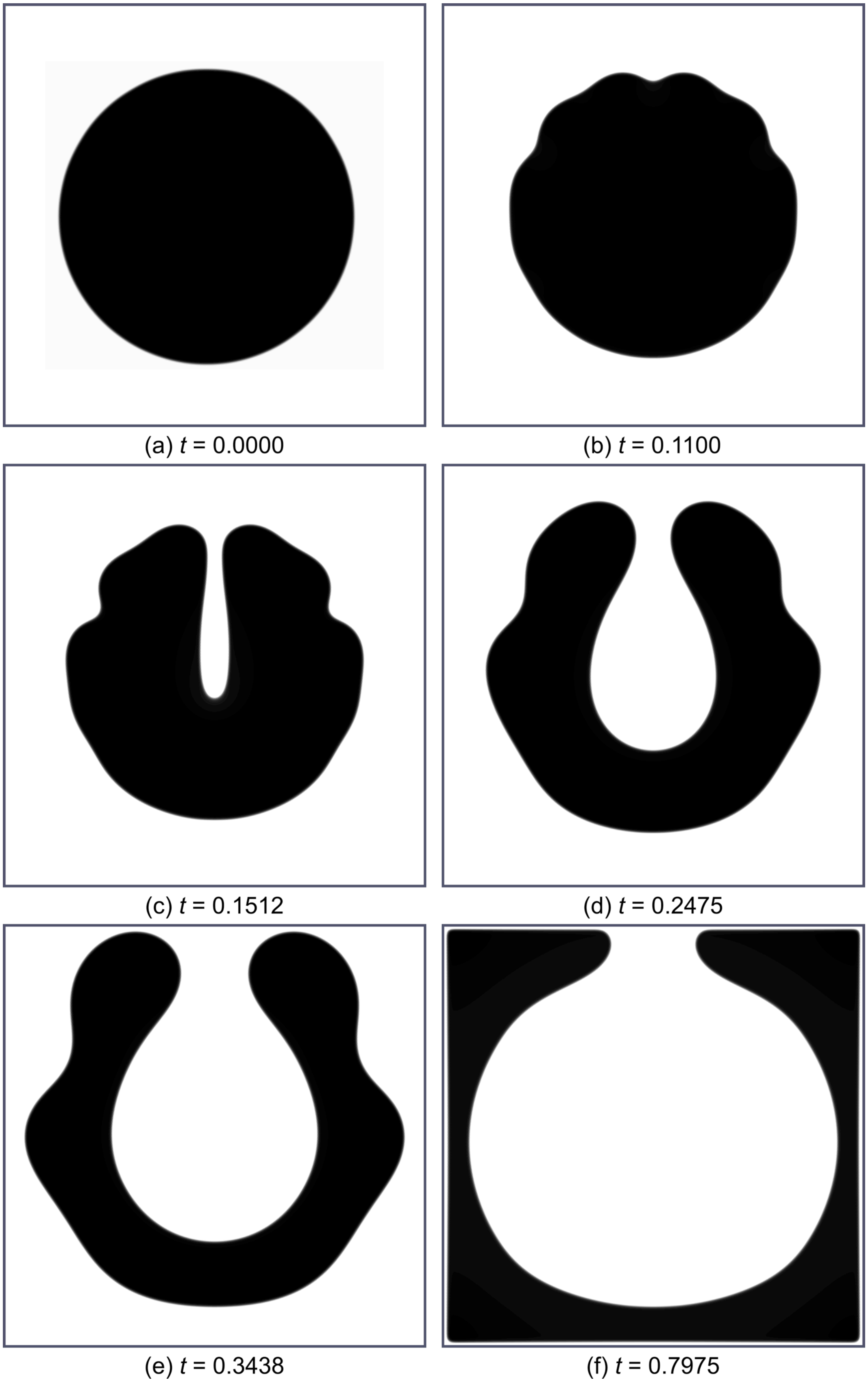

Figure D2. Evolution in pseudo-time t of the gradient flow ($\sigma_1 = -\sigma_2 = 1$, $\sigma_3 = 0$).

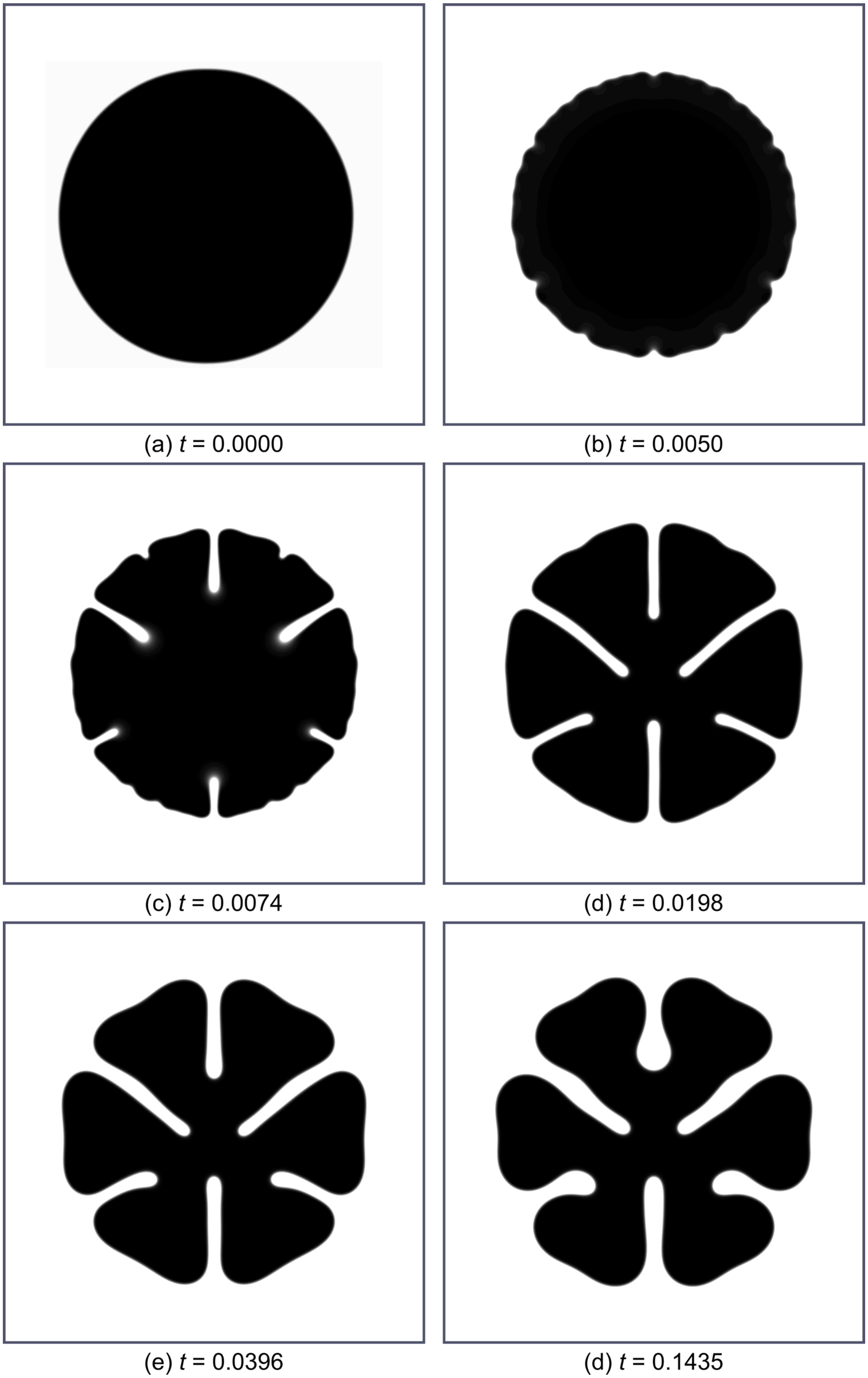

Figure D3. Evolution in pseudo-time t of the gradient flow ($\sigma_1 = 1$, $\sigma_2 = \sigma_3 = 0$).

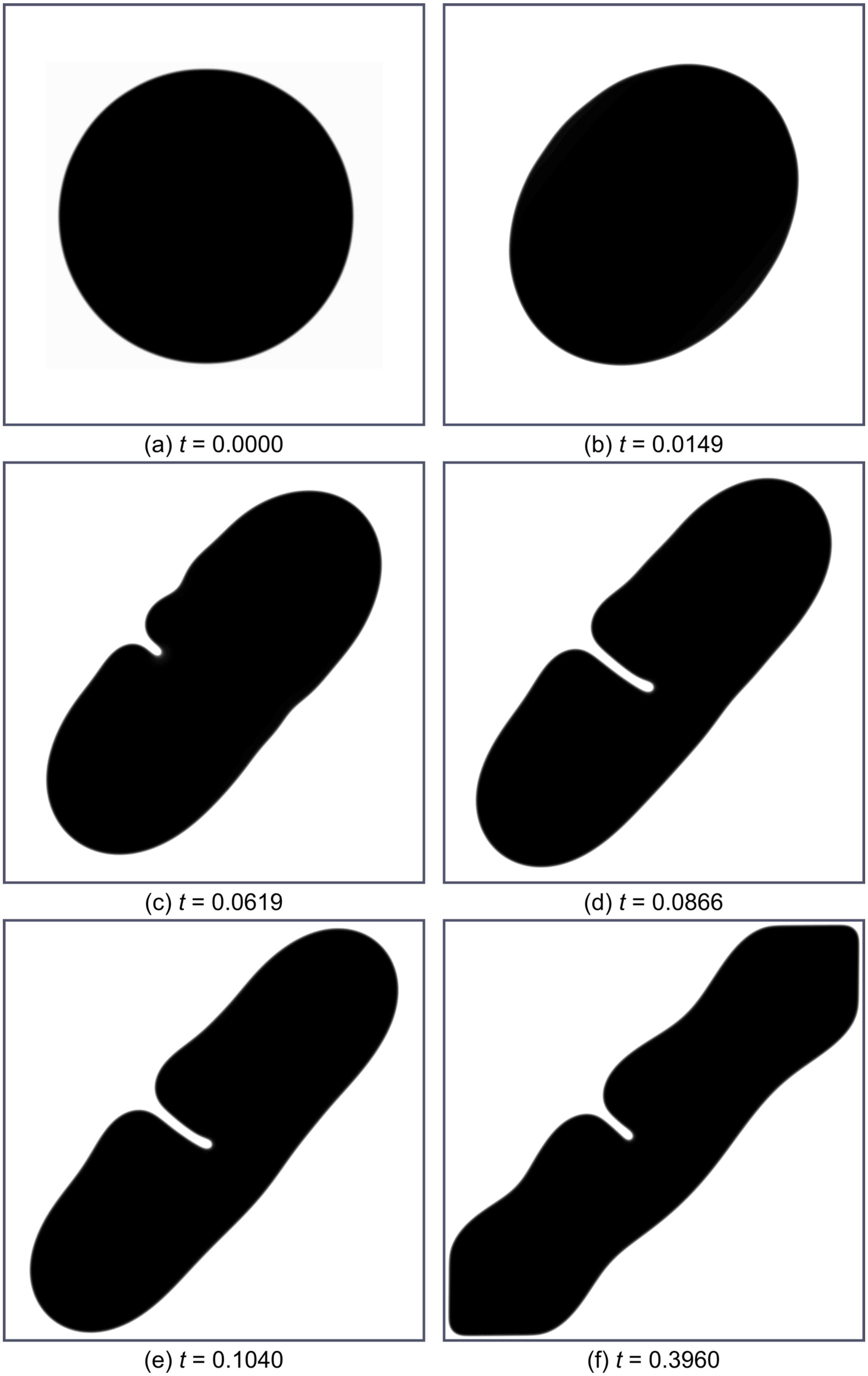

Figure D4. Evolution in pseudo-time t of the gradient flow ($\sigma_1 = \sigma_2 = 1$, $\sigma_3 = 0$).

References

1. Hinze, M., Pinnau, R., Ulbrich, M. & Ulbrich, S. *Optimization with PDE constraints*. No. 23 in Mathematical modelling (Springer Science & Business Media, 2009).

2. Rowe, N., Isnard, S. & Speck, T. Diversity of mechanical architectures in climbing plants: an evolutionary perspective. *J. Plant Growth Regul.* **23**, 108–128 (2004).
